# Automatic Acne Object Detection and Acne Severity Grading Using Smartphone Images and Artificial Intelligence

**DOI:** 10.3390/diagnostics12081879

**Published:** 2022-08-03

**Authors:** Quan Thanh Huynh, Phuc Hoang Nguyen, Hieu Xuan Le, Lua Thi Ngo, Nhu-Thuy Trinh, Mai Thi-Thanh Tran, Hoan Tam Nguyen, Nga Thi Vu, Anh Tam Nguyen, Kazuma Suda, Kazuhiro Tsuji, Tsuyoshi Ishii, Trung Xuan Ngo, Hoan Thanh Ngo

**Affiliations:** 1 Medical AI Co., Ltd., Ho Chi Minh City 700000, Vietnam; hthquan28@gmail.com (Q.T.H.); nhoangphuc.bme@gmail.com (P.H.N.); lexuanhieu131297@gmail.com (H.X.L.); ntlua@hcmiu.edu.vn (L.T.N.); tnthuy@hcmiu.edu.vn (N.-T.T.); dungmaititi@yahoo.com (M.T.-T.T.); bstamhoan@gmail.com (H.T.N.); vunga0501@gmail.com (N.T.V.); ntamanh@medvnu.edu.vn (A.T.N.); 2 School of Biomedical Engineering, International University, Vietnam National University-HCMC, Ho Chi Minh City 700000, Vietnam; 3 University of Medicine and Pharmacy Clinic 1, Ho Chi Minh City 700000, Vietnam; 4 People’s Hospital 115, Ho Chi Minh City 700000, Vietnam; 5 School of Medicine, Vietnam National University-HCMC, Ho Chi Minh City 700000, Vietnam; 6 Rohto Pharmaceutical Co., Ltd., Basic Research Division, Research Village Kyoto, 6-5-4 Kunimidai, Kizugawa, Kyoto 619-0216, Japan; suda@rohto.co.jp (K.S.); ishii@rohto.co.jp (T.I.); 7 Rohto Pharmaceutical Co., Ltd., Regulatory Affairs Promotion Division, 1-8-1 Tatsumi-nishi, Ikuno-ku, Osaka 544-8666, Japan; ktsuji@rohto.co.jp

**Keywords:** deep learning, smartphone image, acne grading, acne object detection

## Abstract

Skin image analysis using artificial intelligence (AI) has recently attracted significant research interest, particularly for analyzing skin images captured by mobile devices. Acne is one of the most common skin conditions with profound effects in severe cases. In this study, we developed an AI system called AcneDet for automatic acne object detection and acne severity grading using facial images captured by smartphones. AcneDet includes two models for two tasks: (1) a Faster R-CNN-based deep learning model for the detection of acne lesion objects of four types, including blackheads/whiteheads, papules/pustules, nodules/cysts, and acne scars; and (2) a LightGBM machine learning model for grading acne severity using the Investigator’s Global Assessment (IGA) scale. The output of the Faster R-CNN model, i.e., the counts of each acne type, were used as input for the LightGBM model for acne severity grading. A dataset consisting of 1572 labeled facial images captured by both iOS and Android smartphones was used for training. The results show that the Faster R-CNN model achieves a mAP of 0.54 for acne object detection. The mean accuracy of acne severity grading by the LightGBM model is 0.85. With this study, we hope to contribute to the development of artificial intelligent systems to help acne patients better understand their conditions and support doctors in acne diagnosis.

## 1. Introduction

Acne is one of the most common skin conditions [1], with acne prevalence reaching 9.38% among the entire world population [2]. Acne occurs owing to blockage or damage of the sebaceous glands and hair follicles. The most common areas affected are the face, chest, neck, shoulders, and back [3]. Acne lesions can be classified into two major types based on their non-inflammatory and inflammatory characteristics. Non-inflammatory lesions include blackheads and whiteheads. Inflammatory lesions include papules, pustules, nodules, and cysts [4]. Acne mainly occurs in adolescents at puberty, affecting 85% of adolescents, and can persist into adulthood [1]. Acne can cause a wide range of effects, from a physical appearance, e.g., scars, to a psychological effect, such as anxiety, poor self-image, lack of confidence, and other negative effects [5]. Improper treatment or treatment delay can lead to physical and mental health damages, which sometimes cannot be restored, e.g., in case of scarring. Approximately 20% of people affected by acne develop severe acne, which can result in scarring if not treated properly [6]. In addition to its impact on individual patients, acne also has a significant impact on the economy. In the United States, the total treatment costs and loss of productivity related to acne have reached $3 billion annually [7]. According to the US Food and Drug Administration (FDA), the average cost per person for acne treatments over a 7-month period is $350 to $3806 [8]. Accurate and timely diagnosis of acne is important for effective acne treatment.

To receive an acne diagnosis, acne patients traditionally have to visit a doctor’s office, where the dermatologist often observes the affected areas by the naked eye or through a dermatoscope. In combination with other types of information, dermatologists give a diagnosis. This process is dependent on the expertise and experience of the dermatologist [9,10]. In addition, owing to the lack of dermatologists in many areas of the world, many patients must travel long distances or wait for a long time before they can see one. Recent advances in smartphone technology and its widespread diffusion, with approximately 3.2 billion people around the world using smartphones [11], are opening doors for many smartphone-based solutions in healthcare [12]. One example is teledermatology, in which patients can receive consultations from a dermatologist at home through a smartphone without a visit to the doctor’s office, thus saving the patient’s time. Teledermatology can increase access to dermatological care for patients, particularly patients living in rural and remote areas. On the other hand, highly accurate, automatic skin image analysis algorithms can potentially help doctors to reduce time and improve accuracy of diagnosis and provide useful information to patients. Developing and integrating these algorithms into dermatology and teledermatology is an active area of research.

Many skin image analysis algorithms have been developed, including algorithms for acne analysis [13,14,15]. However, owing to the complexity of skin lesions, methods based on conventional image processing often do not achieve good results. The advent of deep learning techniques, particularly the convolutional neural network (CNN), has revolutionized the computer vision field in general, and skin image analysis in particular. Several studies have recently been conducted for acne analysis using deep learning to improve the weaknesses of conventional image processing methods.

In 2018, Xiaolei Shen et al. [9] proposed a method to automatically diagnose facial acne based on a CNN. The method was able to distinguish seven classes of acne lesions (blackheads, whiteheads, papules, pustules, modules, cysts, and normal skin). The results showed accuracy of any class > 81%. However, facial images used in Xiaolei Shen et al.’s study were non-smartphone images.

In 2019, Junayed et al. [16] used the AcneNet model based on a deep residual neural network to classify five classes of acne lesions (Closed Comedo, Cystic, Keloidalis, Open Comedo, and Pustular). A total of 1800 images were divided equally between classes with 360 images for each class. The accuracy was over 94% with 99.44% accuracy for the Keloidalis class. However, the images used in Junayed et al.’s study were also non-smartphone images. 

At the end of 2019, Seite et al. reported an artificial intelligence algorithm based on deep learning for facial acne analysis using smartphone images [17]. The method can grade the severity of facial acne based on the Global Evaluation Acne (GEA) scale and identify different types of acne lesions (comedonal lesion, inflammatory lesion, and post-inflammatory hyperpigmentation). The method used a dataset collected from 1072 acne patients, using both iOS and Android smartphones, with a total of 5972 images. The dataset was diverse in terms of skin color and race, with skin images of Asians, Europeans, Africans, and Latinos. The method achieved an F1 score of 84% for inflammatory lesions, 61% for non-inflammatory lesions, and 72% for post-inflammatory hyperpigmentation. However, accuracy in acne severity grading provided by the method was only 68%. 

In 2021, Yin Yang et al. [18] developed another acne assessment algorithm using deep learning for facial acne severity grading according to the Chinese Guidelines. A dataset composed of 5871 clinical images of 1957 patients was collected using Fujifilm and Canon cameras. The method had three steps: preprocessing image data to remove interference from the eyes, nose, and mouth areas; classifying acne lesions using an Inception-V3 network; and, finally, evaluating the model performance in patients with acne vulgaris. The results showed an average F1 score value of 0.8 for the deep learning model and a Kappa coefficient (coefficient for evaluating the correlation between the deep learning model and the dermatologists) of 0.791. Except for one study [17], most of the above studies used non-smartphone images and only focused on either the task of acne severity grading or acne object detection, but not both.

In this study, we developed an AI system called AcneDet based on deep learning and machine learning that can automatically analyze facial smartphones images for both tasks: (1) detecting acne lesion objects of four types: blackheads/whiteheads, papules/pustules, nodules/cysts, and acne scars; and (2) grading acne severity based on the IGA scale [19]. Output of the acne object detection step was used as input to the acne severity grading step. The data used in this study include 1572 images collected by both iOS and Android smartphones. The data were then labeled by four dermatologists and used for AI training. 

## 2. Materials and Methods

### 2.1. Dataset

#### 2.1.1. Data Collection and Labeling

Data were retrieved from a database of a mobile application called Skin Detective, developed by our team, that is available on both iOS and Android smartphones. Users agreed with the app’s terms and conditions before using the app. Only with user agreement, users’ facial images were stored in the app’s database for AI training. For each user that agreed to share facial images for AI training, three selfie facial images taken at three different angles, i.e., the front, left, and right angles, were collected. During image capturing, distance from the user’s face to smartphone camera and lighting condition were checked by the mobile application. When the distance was within ~20 cm and lighting condition was sufficient, the app would inform the user to capture an image. All captured images were later manually checked for quality. Low quality images were removed. The remaining, a total of 1572 de-identified images of people of various race and skin color, were included in this study. The average size of the images was 720 pixel × 1280 pixel. The images were labeled by four dermatologists (two juniors and two seniors). The dermatologists used LabelBox software to label images. There were two main labeling tasks. One was to draw rectangular bounding boxes to mark the location and type of acne lesions. Four types of acne were labeled, including blackheads/whiteheads, papules/pustules, nodules/cysts, and acne scars. In this task, each image was first labeled using a junior dermatologist. A senior dermatologist then reviewed and corrected the labeling if necessary. Another labeling task was to grade the acne severity for each image based on the results of the first labeling task. Acne severity was graded based on the IGA [19] scale: 0, clear; 1, almost clear; 2, mild; 3, moderate; and 4, severe. Like the first labeling task, the acne severity of each image was first graded by a junior dermatologist and then reviewed and corrected, if necessary, by a senior dermatologist. 

#### 2.1.2. Data Statistics

A total of 41,859 acne lesions were labeled among 1572 images. Among them, acne scars are the most common, with 23,214 (55.46%), and nodular lesions are the least common, with 282 (0.67%). The number and percentage of each acne lesion type are detailed in Table 1.

In terms of acne severity, grade 1 is the most prevalent, with 56.18%, and grade 4 is the least, with 2.16%. The distribution of acne severity grades is shown in Table 2.

**Figure 1:**
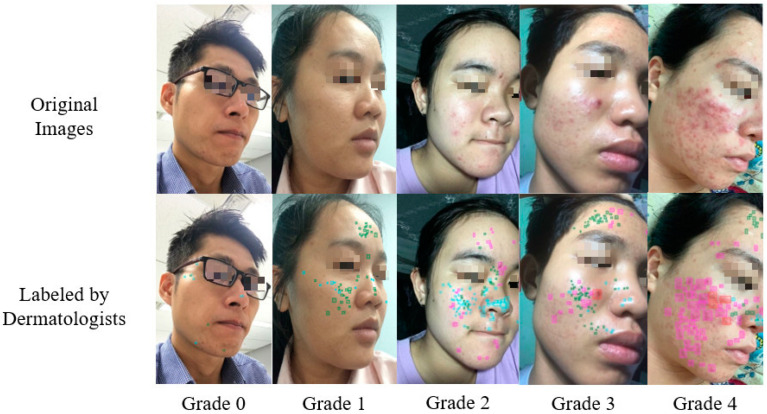
From the original images, dermatologists labeled acne lesions (blackheads/whiteheads, papules/pustules, nodules/cysts, and acne scars) using bounding boxes and graded the acne severity using the IGA scale: grade 0, clear; grade 1, almost clear; grade 2, mild; grade 3, moderate; and grade 4, severe.

Figure 1 shows acne lesions that were labeled by dermatologists. The top row shows the original images of the patients captured by smartphones. The bottom row shows the corresponding images labeled by dermatologists. For each image, the dermatologists marked the locations of the acne lesions using bounding boxes and gave an acne severity grade using the IGA scale. Each acne lesion type has a distinct bounding box color: cyan for blackheads/whiteheads, pink for papules/pustules, red for nodules/cysts, and green for acne scars.

### 2.2. IGA Scale

The IGA scale was recommended by the FDA to be a static, qualitative evaluation of overall acne severity. It has five levels ranging from grade 0 to grade 4, with grade 0 being clear; grade 1, almost clear; grade 2, mild; grade 3, moderate; and grade 4, severe (Table 3).

### 2.3. Methods

#### 2.3.1. Overall Model Architecture

In terms of the overall architecture, the system includes two models for two different tasks:

1. Acne object detection model: determine the location and type of acne lesions.
2. Acne severity grading model: grade the overall acne severity of the input image using the IGA scale.

**Figure 2:**
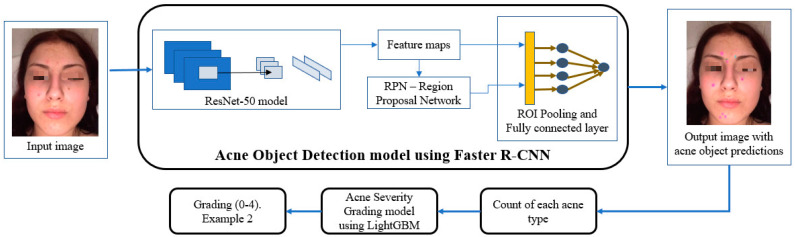
Pipeline of acne lesion object detection and acne severity grading system with two main steps: acne object detection and acne severity grading. The output of the acne object detection model was used as input to the acne severity grading model.

The architecture of the system is shown in Figure 2. The output of the acne object detection model, more specifically, the numbers of each acne type, was used as input for the acne severity grading model. In this way, our system mimics the acne severity grading process of dermatologists, in which the number of blackheads/whiteheads, papules/pustules, nodules/cysts, and acne scar lesions are first estimated, followed by applying the IGA scale rules shown in Table 3. An advantage of this approach is that the result is easy to interpret. Acne objects are detected and marked by bounding boxes. Different acne types have different bounding box colors. Acne severity is graded based on the numbers of each acne type. Therefore, the acne severity grade prediction output by the system can be easily explained, thus avoiding the black box issue commonly found in CNN-based classifiers.

***Acne object detection model***

We chose the Faster R-CNN architecture [20] with the ResNet50 backbone to build our acne object detection model. The model was trained for 13,000 epochs on an NVIDIA GTX 2080, using an Adam optimizer with a training time of 2 weeks. A Faster R-CNN is one of the state-of-art object detection architectures.

***Acne severity grading model***

We built our acne severity grading model based on the LightGBM algorithm [21], a tree-based machine learning algorithm. The model input, i.e., the numbers of each acne type, comes from the output of the acne object detection model. LightGBM is a fast, high-performance machine learning model that has performed well on various machine learning competitions.

#### 2.3.2. Training Model

**Figure 3:**
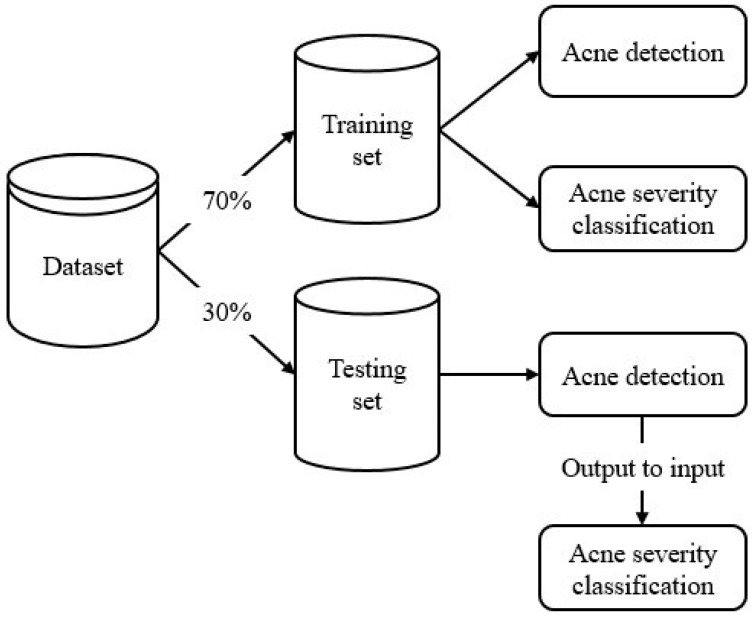
Data were divided into a ratio of 70:30 for training and testing.

We divided the dataset into two sets, as shown in Figure 3: a training set and a testing set, with a ratio of 70:30. During the training phase, because information on acne objects, counts, and severity grades were all available, both models were trained in a parallel fashion on the training data until convergence. However, during the testing phase, the output of the acne object detection model was used as the input for the acne severity grading model. The acne severity grading model in turn output the acne severity grade based on the IGA scale.

***Evaluation metrics***

We measured the model performance using two main metrics: the mean Average Precision (mAP) for the Acne object detection model and the area under the receiver operating characteristic (ROC) curve (AUC) for the acne severity grading model.

## 3. Results

### 3.1. Acne Object Detection

**Figure 4:**
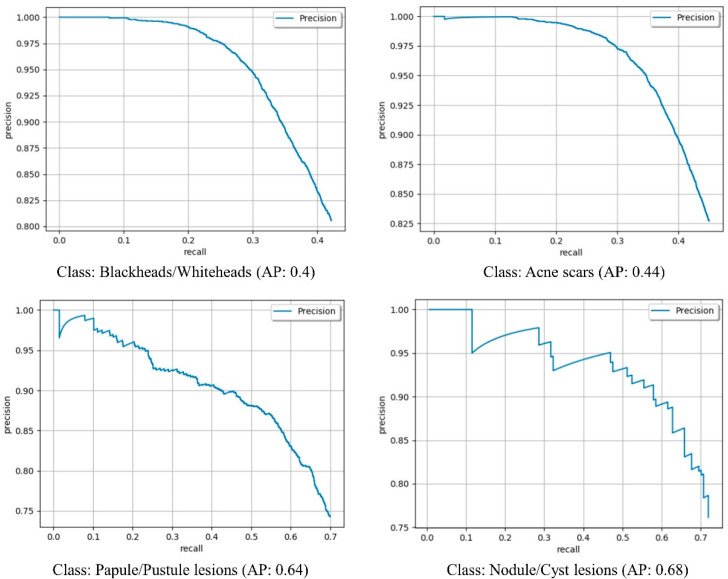
Precision–recall curve of object detection of each acne type.

To evaluate the performance of the acne object detection model in detecting acne objects, we used the Average Precision (AP) for each acne type and the mean Average Precision (mAP) for all four acne types. Figure 4 details the precision–recall curve for each acne type. AP for each acne type and mAP for all four acne types are shown in Table 4. As shown, AP for nodule/cyst lesions was the highest at 0.68 and blackhead/whitehead lesions were the lowest at 0.4. For all four acne types, we achieved a mAP of 0.54. 

### 3.2. Acne Severity Grading

**Figure 5:**
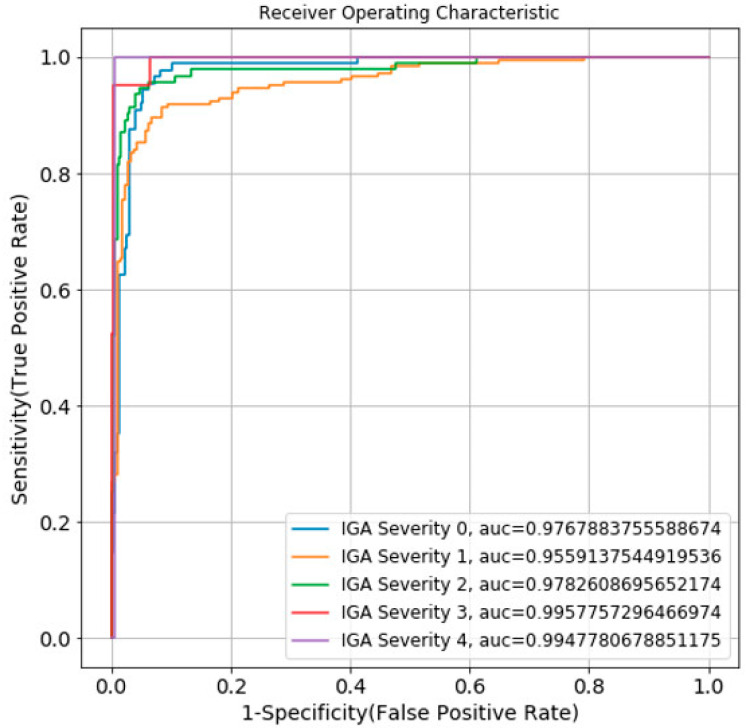
ROC–AUC diagram of the acne severity grading model.

**Figure 6:**
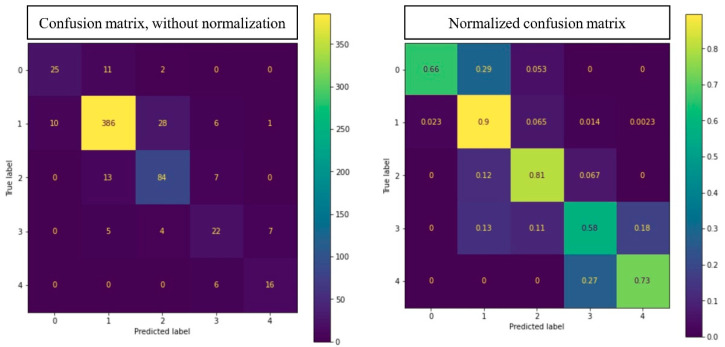
Confusion matrix with and without normalization on test set.

To evaluate the performance of the acne severity grading model, we used the AUC. The ROC curve and AUC of each acne severity grade of 0–4 in the IGA scale area are shown in Figure 5. A normalized confusion matrix and non-normalized confusion matrix of the acne severity grading model are shown in Figure 6. 

**Figure 7:**
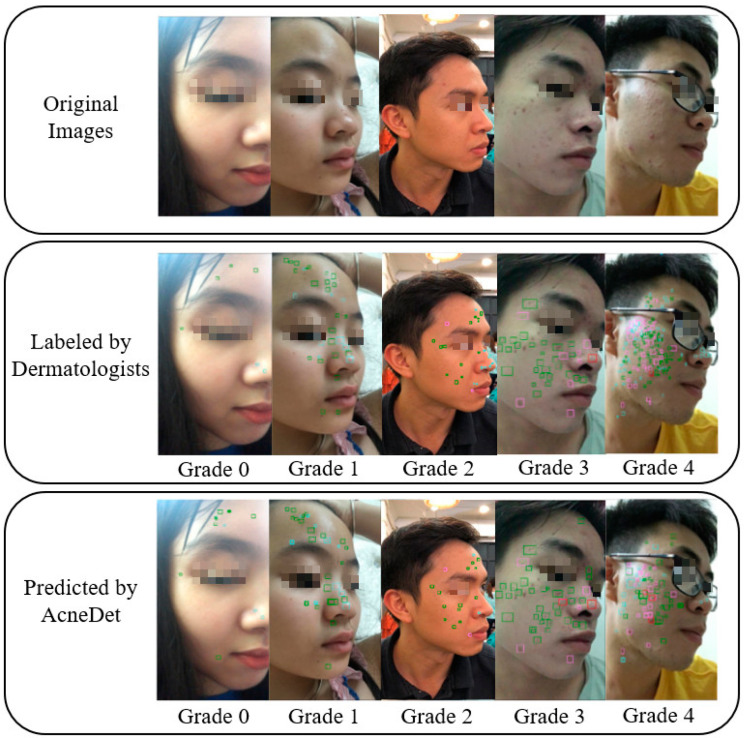
Comparison of predictions by AcneDet with ground truths labeled by dermatologists.

In addition to the AUC, we also calculated the accuracy, precision, recall, and F1 (Table 5). The average accuracy for the five grades was 0.85. Figure 7 shows some examples of input images, the ground truth labeled by dermatologists, and the prediction by our AcneDet system. The system outputs two main predictions: (1) the location and type of acne objects in the image; and (2) the acne severity grade of the image. The accuracy in detecting acne objects and the grading of acne severity both contribute to the overall performance of AcneDet.

## 4. Discussion

We believe that skin image analysis algorithms will play an important role in future dermatology, where dermatologists will likely be supported by AI systems during the diagnosis and treatment processes. Patients will also benefit from information provided by highly accurate skin image analysis algorithms. Acne is one of the most common skin conditions, affecting 9.38% of the world population, and can cause serious effects, including psychological effects and effects on quality of life. In this study, we developed an AI system called AcneDet that can automatically detect acne lesion objects and grade acne severity with a high level of accuracy.

For the acne lesion object detection task, we used a Faster R-CNN architecture to build our model trained on a dataset of 41,859 acne objects. The Faster R-CNN performed reasonably well, with a mAP for all four acne types of 0.54. This mAP is higher than the previous results of Kuladech et al. [10] and Kyungseo Min et al. [22]. Kuladech’s study trained an R-FCN model on a dataset of 15,917 acne images and achieved a mAP of 0.283 for four acne types. Kyungseo Min’s study trained an ACNet model that was composed of three main components, composite feature refinement, dynamic context enhancement, and mask-aware multi-attention on the ACNE04 dataset with 18,983 acne images. They achieved a mAP of 0.205 for acne object detection and did not classify acne type. A comparison between our approach and the above methods is shown in Table 6.

It is noteworthy that our AI system was trained on a dataset with a larger number of acne objects. Regarding the acne types, the number of acne types in our study is the same as in Kuladech’s study, i.e., four, and is higher than in Kyungseo Min’s study. Our high mAP in acne object detection, in combination with our two-model and two-stage approach, results in the high accuracy of our acne severity grading. Note that, although the number of nodule/cyst lesions in our dataset is extremely small (only 0.67% of the total number of acne lesions), our model was able to detect nodule/cyst lesions with a mAP of 0.68. This can be explained by the fact that nodule/cyst lesions often have an extremely distinct color (usually red) and a substantially larger size. Thus, our model could learn to recognize them better. By contrast, whereas the number of acne scars accounted for 55.46% of the total number of acne objects, the mAP was only 0.44. We attribute this to the fact that most of the acne scars are small with a color not much different from the surrounding skin. Therefore, although the training examples of acne scars were abundant, the model had a hard time learning to recognize them. We believe the same applied to blackhead/whitehead lesions, which accounted for 37.47% of the total number of acne objects, but had the lowest mAP of 0.4.

To grade the severity of the acne, we used the output of the acne object detection model, specifically the number of each acne type, as input for the LightGBM-based acne severity grading model. Using this approach, we achieved an average accuracy of 0.85 for all five grades. The accuracy of our acne severity grading model is compared with the accuracy of previous studies in Table 7. The results show that our model achieved a higher accuracy than that of previous studies. More specifically, in comparison to the 0.67 accuracy reported by Ziying Vanessa et al., who also used the IGA scale [23], our accuracy of 0.85 is significantly higher. There is a significant imbalance in our dataset in regard to the number of grade 3 (moderate) and grade 4 (severe) images, which are quite low at 83 (5.28%) and 34 (2.16%), respectively. This reflects the scarcity of these two grades within the population. Since these two grades can have serious effects on patients, accurately grading them is important. Our system achieved acceptable F1 scores for these two grades, with grades 3 and 4 having F1 scores of 0.60 and 0.74, respectively. In the future, we plan to collect more images of grades 3 and 4 to further improve grading accuracy for these two grades.

Notably, the images used in our study were collected through smartphones. Therefore, they are lower in quality and less clinically informative than images obtained through digital cameras or a dermatoscope, making their analysis more challenging. However, we were able to achieve a mAP of 0.54 and an accuracy of 0.85, which are higher than those of previous studies, which is a good foundation for future studies. Given the high number of people with acne, the shortage of dermatologists in many parts of the world, and the popularity of smartphones, an AI-powered smartphone app that can quickly and accurately analyze smartphone-captured facial images and help users to understand their acne conditions better, e.g., severity and acne type, is potentially useful. In the case of high grade/severe acne, the app could recommend and connect acne patients with remote dermatologists for teledermatology. In the case of low grade/mild acne, the app could recommend skincare products, diet, and lifestyle adjustments, etc. so that acne patients can achieve an acne-clear or near acne-clear skin condition. Through this study, we hope to contribute to the development of acne analysis algorithms to help acne patients better understand their conditions and support doctors in acne diagnosis.

## 5. Conclusions

In this study, we developed an AI system called AcneDet for acne object detection and acne severity grading, using facial images captured by smartphones. The AcneDet system consists of two models for two different tasks: an acne object detection model using Faster R-CNN-based deep learning and an acne severity grading model based on LightGBM machine learning. Four types of acne could be detected: blackheads/whiteheads, papules/pustules, nodules/cysts, and acne scars. The test results show that the acne object detection model achieved a mAP of 0.54. The output of the acne object detection model was used as input for the acne severity grading model, which achieved an accuracy of 0.85. In the future, we plan to collect more data to improve the mAP and accuracy of the system, and apply semi-supervised and unsupervised learning techniques to reduce the labeling workload. In addition, we are also interested in developing AIs that could perform other tasks such as identifying acne patients that need immediate initiation of treatment to avoid scarring. We believe that such AIs would need to consider other factors such as age and sex, etc., in addition to facial images, to make accurate predictions.

## Figures and Tables

**Table 1:** Statistics of different types of acne.

Type of Acne	Number of Acne Type	Ratio (%)
Blackheads/Whiteheads	15,686	37.47
Acne scars	23,214	55.46
Papules/Pustules	2677	6.4
Nodular/Cyst lesions	282	0.67
Total	41,859	100

**Table 2:** Statistics of different acne severity grades based on the IGA scale.

IGA Scale of Acne Severity Grade	Number of Images	Ratio (%)
0	211	13.42
1	883	56.18
2	361	22.96
3	83	5.28
4	34	2.16
Total	1572	100

**Table 3:** Detailed description of the IGA scale [19].

Grade	Description
0	Clear skin with no inflammatory or non-inflammatory lesions
1	Almost clear; rare non-inflammatory lesions with no more than one small inflammatory lesion
2	Mild severity; greater than Grade 1; some non-inflammatory lesions with no more than a few inflammatory lesions (papules/pustules only, no nodular lesions)
3	Moderate severity; greater than Grade 2; up to many non-inflammatory lesions and may have some inflammatory lesions, but no more than one small nodular lesion
4	Severe; greater than Grade 3; up to many non-inflammatory lesions and may have some inflammatory lesions, but no more than a few nodular lesions

**Table 4:** Average Precision (AP) for each acne type and mean Average Precision (mAP) for all four acne types.

Type of Acne	AP
Blackheads/Whiteheads	0.4
Acne scars	0.44
Papule/Pustule lesions	0.64
Nodular/Cyst lesions	0.68
mAP for all four acne types	**0.54**

**Table 5:** Precision, recall, F1 score, and accuracy of acne grading model.

Grade of IGA Scale	Precision	Recall	F1
0	0.77	0.63	0.70
1	0.92	0.90	0.91
2	0.72	0.77	0.75
3	0.60	0.61	0.60
4	0.65	0.87	0.74
Accuracy	0.85		

**Table 6:** Comparison of the mAP in detecting acne objects obtained in our study and in previous studies.

Authors	Acne Types	Number of Acne	Model	mAP
Kuladech et al. [10]	Type I, Type III, Post-inflammatory erythema, Post-inflammatory hyperpigmentation	15,917	Faster R-CNN, R-FCN	Faster R-CNN: 0.233 R-FCN: 0.283
Kyungseo Min et al. [22]	General Acne (not classification)	18,983	ACNet	0.205
**Our method**	Blackheads/Whiteheads, Papules/Pustules, Nodules/Cysts, and Acne scars	41,859	Faster R-CNN	**0.540**

**Table 7:** Comparison of accuracy in grading acne severity obtained through our study and in previous research.

Authors	Acne Severity Scale	Number of Images	Model	Accuracy
Sophie Seite et al. [17]	GEA scale	5972		0.68
Ziying Vanessa et al. [23]	IGA scale	472	Developed based on DenseNet, Inception v4 and ResNet18	0.67
Yin Yang et al. [18]	Classified according to the Chinese guidelines for the management of acne vulgaris with 4 severity classes	5871	Inception-v3	0.8
**Our method**	IGA scale	1572	LightGBM	**0.85**

## Data Availability

The data presented in this study are not publicly available due to privacy issues.
